# Quantitative evaluation using single-photon emission computed tomography with acetazolamide is reliable for preoperative evaluation before cardiac surgery in severe carotid intracranial artery stenotic and/or occlusive disease: a case report

**DOI:** 10.1186/s13019-019-0961-4

**Published:** 2019-07-23

**Authors:** Eiki Tayama, Ryusuke Mori, Tomohiro Ueda, Ken-ichi Imasaka, Yukihiro Tomita, Shigeki Morita

**Affiliations:** Department of Cardiovascular Surgery, Clinical Research Institute, National Hospital Organization, Kyushu Medical Center, 1-8-1 Jigyohama, Chuo-ku, Fukuoka, 810-8563 Japan

**Keywords:** Carotid and intracranial artery stenotic and/or occlusive disease (CIAD), Single-photon emission computed tomography (SPECT), Cerebral hemodynamics, Off-pump coronary artery bypass grafting (OPCAB)

## Abstract

**Background:**

Severe carotid and intracranial artery stenosis disease (CIAD) is major risk for perioperative stroke in coronary artery bypass grafting. Then, preoperative risk assessment is quite important.

**Case presentation:**

A 58-years old Japanese woman with bilateral carotid stenosis and bilateral middle cerebral artery occlusion was suffered from worsening effort angina due to severe three coronary vessel disease. Magnetic resonance imaging angiography demonstrated severe carotid and intracranial vessel stenosis. Selective carotid/cerebral angiography also showed severe stenosis and delayed filling of the right internal carotid artery and moderate stenosis of the left internal carotid artery, with occlusion of the bilateral middle cerebral arteries. However, quantitative evaluation with brain perfusion, single-photon emission computed tomography (SPECT) with acetazolamide showed depleted cerebral perfusion volume and vascular responses, particularly in the left middle cerebral artery area. However, both sides of MCA reserve cerebral blood flow was maintained at > 34 ml/100 g/min. So, we finally considered that her cerebral perfusion reserve was maintained a certain level and could tolerate open heart surgery. Then, she underwent off-pump coronary artery grafting. Before sternotomy, prophylactic intra-aortic balloon pump support was used to minimize possible perioperative stroke. As a result, hemodynamic status and brain regional oxygen saturation were stable throughout the operation, and recovered uneventfully.

**Conclusions:**

Preoperative quantitative evaluation using brain perfusion SPECT with acetazolamide is useful in assessing hemodynamic cerebrovascular risk in patients with severe obstructive CIAD. Off pump coronary artery bypass grafting with intra aortic balloon pump assist is a good option for prevention of cerebrovascular morbidity in ischemic heart disease with severe CIAD.

## Background

Although embolism is the major cause for perioperative stroke, carotid and intracranial artery stenotic and/or occlusive disease (CIAD) is known to increase perioperative stroke and mortality risk after coronary artery bypass grafting (CABG) [1, 2]. Therefore, risk assessment and operative strategy should be carefully considered in such cases. We report a patient with bilateral carotid stenosis and bilateral middle cerebral artery occlusion who underwent coronary artery revascularization without any complications. Quantitative evaluation with brain perfusion, single-photon emission computed tomography (SPECT) with acetazolamide was useful for assessment of perfusion reserve in this patient with severe CIAD [3–5].

## Case presentation

A 58-year-old Japanese woman was admitted with 6 months of recently worsening effort angina. She had no history of previous stroke, but had been treated for diabetes mellitus and hypertension for 7 years and was currently controlled, with glycosylated hemoglobin and blood pressure in normal range. Electrocardiography showed abnormal Q waves in II, III, and aVF, and downsloping ST-segment depression in V4 to V6. Echocardiography showed severe left ventricular anteroseptal hypokinesis and decreased ejection fraction of 38% without mitral regurgitation. Coronary angiography revealed total occlusion of proximal right coronary artery #2, tandem stenosis (75 and 90%) of the left anterior descending coronary artery, and 75% stenosis of the left circumflex coronary artery. Unfortunately, this 3-vessel coronary disease was considered inappropriate for percutaneous coronary intervention due to multiple long stenotic lesions, the patient was referred for CABG.

**Fig. 1:**
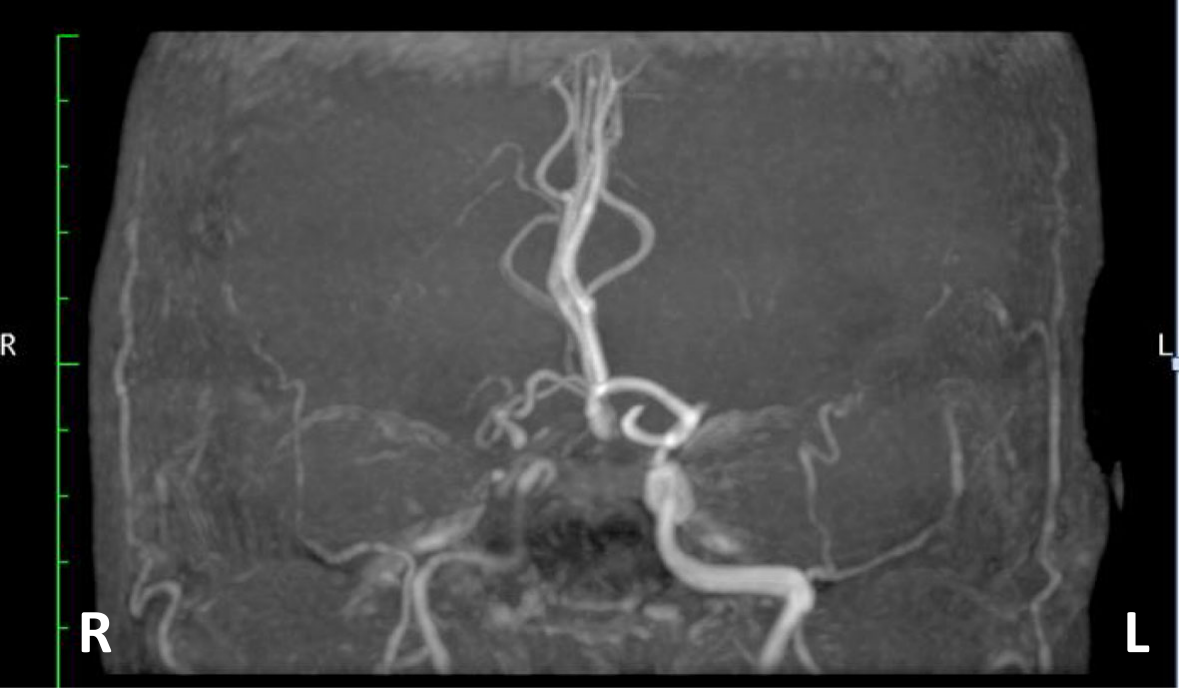
Magnetic resonance imaging (MRI) angiography. MRI demonstrated stenosis in bilateral internal carotid arteries, with occluded and poorly visible bilateral middle cerebral arteries

**Fig. 2:**
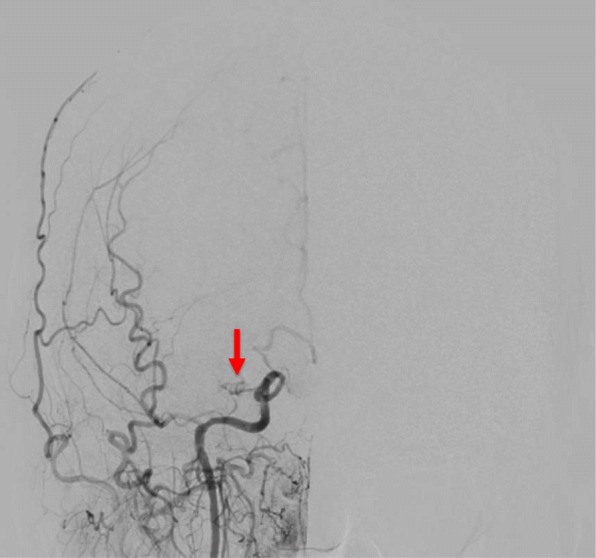
Selective internal carotid artery angiography (Right). Severe stenosis with filling delay in right internal carotid artery. Right middle cerebral artery was occluded (arrow) and showed slight contrast with a filling delay through the anterior communicating artery

**Fig. 3:**
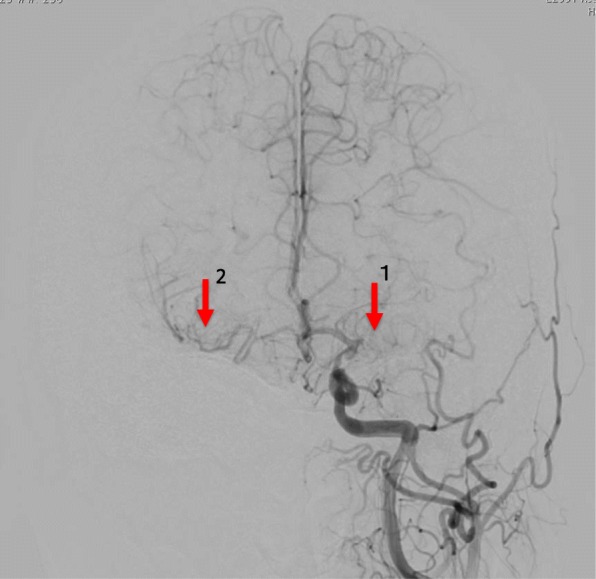
Selective internal carotid artery angiography (Left). Moderate stenosis of the left internal carotid artery and total occlusion of the left middle cerebral artery (arrow 1). Slight collateral circulation (arrow 2) from the left internal carotid artery, external carotid artery, and vertebral artery to the middle cerebral artery territory

**Fig. 4:**
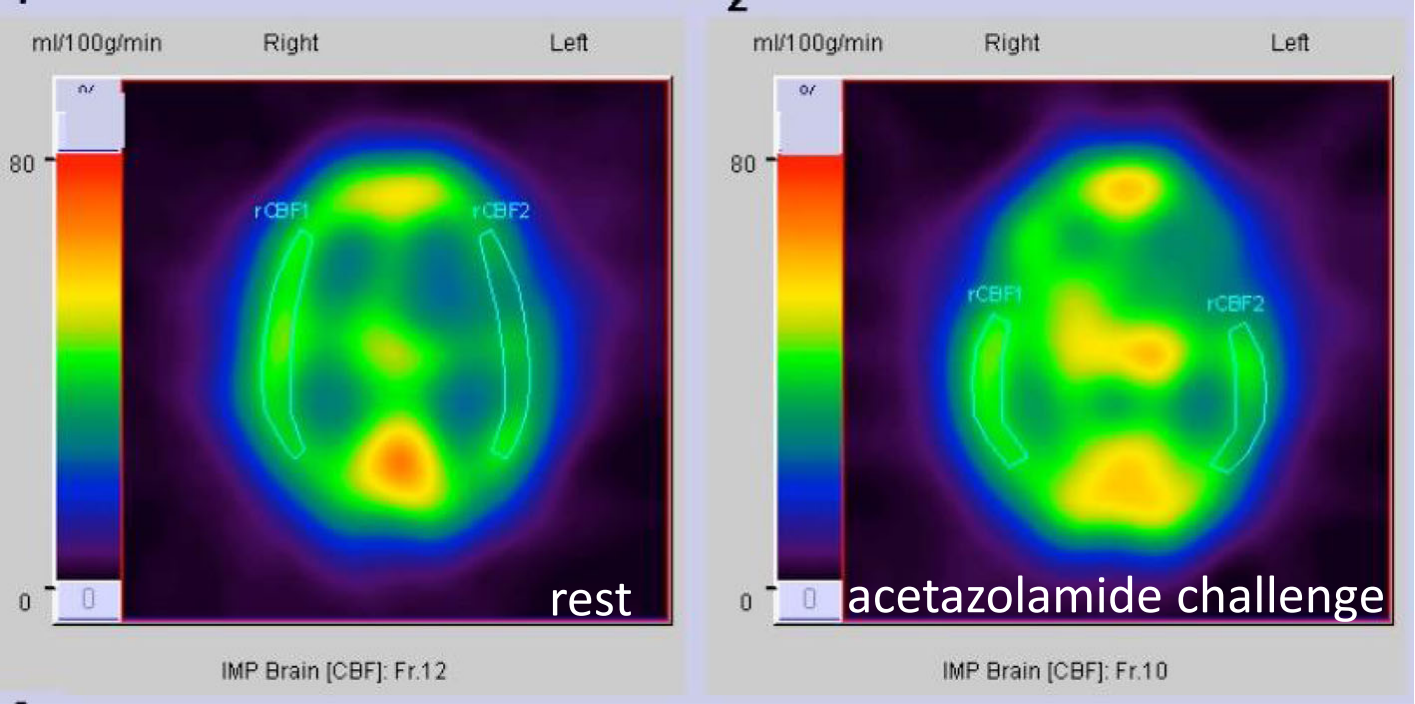
Brain Perfusion SPECT (123I-IMP). Preoperative SPECT (123I-IMP) revealed significant reduction in cerebral blood flow at rest and reactivity to acetazolamide, particularly in the left MCA territory (rest and acetazolamide). SPECT: single-photon emission computed tomography. 123I-IMP: N-isopropyl-p-[123I]iodoamphetamine

However, magnetic resonance imaging (MRI) angiography demonstrated severe carotid and intracranial vessel stenosis (Fig. 1). Selective carotid/cerebral angiography also showed severe stenosis and delayed filling of the right internal carotid artery (ICA) and moderate stenosis of the left ICA, with occlusion of the bilateral middle cerebral arteries (MCAs). The bilateral distal MCAs were slightly enhanced with delayed filling via collaterals from the external carotid arteries and vertebral artery (Figs. 2, 3). SPECT N-isopropyl-p-[123I]iodoamphetamine (123I-IMP) with acetazolamide showed depleted cerebral perfusion volume and vascular responses, particularly in the left MCA area. However, bilateral MCA reserve cerebral blood flow (rCBF) was maintained at > 34 ml/100 g/min (Fig. 4 and Table 1). Fortunately, the ascending aorta and arch were intact, without calcification or atherosclerotic debris on CT.

**Table 1:** Quantitative evaluation of brain perfusion using SPECT (123I-IMP) with acetazolamide

	MCA 1		MCA 2		Cerebellum	
	Rt	Lt	Rt	Lt	Rt	Lt
Rest: reserve cerebral flow (ml/100g/min)	34	36	33	35	38	39
Acetazolamide: rCBF (ml/100g/min)	40	34	41	36	57	66
Vascular Response(%)	18	-5	24	3	50	69

*SPECT* single-photon emission computed tomography

123I-IMP: N-isopropyl-p-[123I]iodoamphetamine

*MCA* middle cerebral artery

**Fig. 5:**
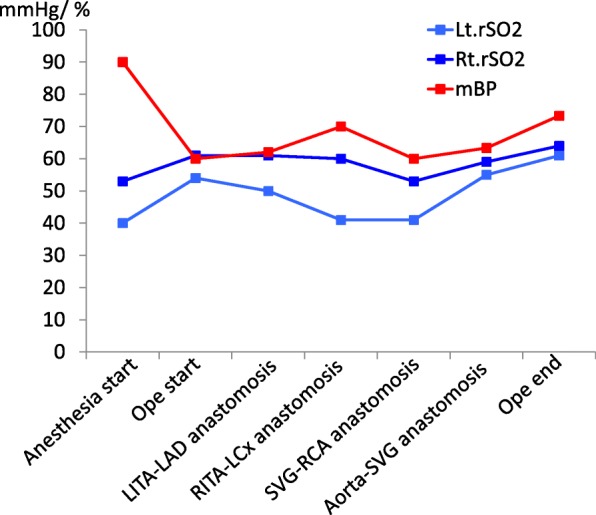
Blood pressure and brain regional oxygen saturation during OPCAB. Throughout the operation, hemodynamic status and brain regional oxygen saturation were stabilized, even during heart displacement. rSO_2_: brain regional oxygen saturation. mBP: mean blood pressure. LITA: left internal thoracic artery. LAD: left anterior descending coronary artery. RITA: right internal thoracic artery. SVG: saphenous vein graft. RCA: right coronary artery. LCx: left circumflex coronary artery

Despite severe CIAD, we considered that the patient could tolerate open heart surgery with appropriate perioperative circulatory management, without need for preoperative cerebral perfusion intervention. She underwent off-pump CABG (OPCAB: left internal thoracic artery to left anterior descending artery #8, right internal thoracic artery to left circumflex artery #14, and saphenous vein graft to right coronary artery #4). Before sternotomy, prophylactic intra-aortic balloon pump (IABP) support was used to minimize possible perioperative stroke. For proximal saphenous grafting to the ascending aorta, we used the Enclose II anastomosis assist device (Novare Surgical Systems, Inc., CA, USA). As a result, hemodynamic status and brain regional oxygen saturation were stable throughout the operation, even during displacement of the heart for posterior and inferior anastomosis (Fig. 5). The postoperative course was uneventful; weaning from both the respirator and IABP was performed on postoperative day 1, with discharge on postoperative day 17 without any complications. The patient has remained stable for more than 3 years.

## Discussion

While CABG has played a significant role in revascularization for ischemic heart disease, neurological complications have been a primary concern. In particular, CIAD increases perioperative stroke and mortality risk [1, 2].

Once CIAD is suspected on carotid ultrasound, CT, or MRI, functional evaluation is essential. As a functional test of brain circulation, SPECT is the most useful method for quantification of cerebral blood flow (CBF). Brain perfusion SPECT, particularly with use of acetazolamide, is considered capable of detecting hemodynamic compromise [3–5]. With the injection of acetazolamide, perfusion is increased in areas where the cerebral perfusion reserve is preserved, whereas areas with decreased cerebral perfusion reserve show no increase or decrease in perfusion because of the intra-cerebral steal phenomenon [3]. Reduced regional cerebral blood flow (< 80% of normal cerebral blood flow) and reduced regional cerebral reactivity (< 10% of regional cerebral reactivity) are diagnosed as having impaired cerebral perfusion reserve (misery perfusion) [3]. In patients with misery perfusion, excessive falls in blood pressure and systemic hemodynamic disturbances lead to hemodynamic ischemic stroke [6].

An our previous report assessed obstructive CIAD in 88 patients of open heart surgery cases using brain perfusion SPECT with acetazolamide [4]. In that study, unacceptably depleted cerebral perfusion reserve was defined as rCBF in the ipsilateral MCA ≤34 ml/100 g/min, with a reserve cerebral blood response of ≤10%, based on the Japanese EC-IC bypass trial (JET study) [7]. An impaired cerebral perfusion reserve was identified in 1 (1.1%) patients. This patient underwent prophylactic superficial temporal artery to MCA anastomosis 1 month before CABG. Subsequently, the patient underwent conventional CABG, without experiencing perioperative stroke. Seven (1.4%) patients died in-hospital mortality and 5 (1.0%) experienced embolic perioperative stroke due to paroxismal atrial fibrillation. However, no patients experienced perioperative “haemodynamic” ischaemic stroke, if cerebral reserve was acceptable range evaluated by the SPECT.

According to this definition, open heart surgery was judged to be acceptable in the present case, as long as appropriate hemodynamic status was maintained. We would like to emphasize that quantitative evaluation using brain perfusion SPECT with acetazolamide is very useful for risk assessment in CIAD patients. If cerebral perfusion reserve is judged as lower than safe limit, some preoperative prophylactic craniocervical intervention may be crucial, such as carotid artery stenting, carotid endarterectomy, and superficial temporal artery to middle cerebral artery bypass.

However, careful patient selection is essential for application of an acetazolamide challenge, because serious adverse effects can occur, such as congestive heart failure or acute pulmonary edema. It is important to apply it only to patients who really need it. In addition, carefully monitor the clinical course, and take appropriate care promptly for possible complications are essential. On the other hand, there are no alternative test for SPECT with acetazolamide, which reproducible and having enough clinical experience.

Even if cerebral circulation reserve is adequate for open heart surgery, cerebral hypoperfusion may occur due to hypotension and declining cardiac output. This may result in diminished washout of embolic materials from blood vessels in the brain. This phenomenon is particularly likely in watershed areas along the boundaries of major arterial territories, predisposing to ischemia in these regions [8]. In addition, the risk of ischemic stroke distal to obstructive CIAD is associated with impaired cerebral autoregulation [6, 9].

Whether OPCAB or on-pump CABG is superior remains controversial. Several randomized studies comparing OPCAB with conventional on-pump CABG failed to show an advantage with OPCAB regarding stroke [10–12]. On the other hand, large retrospective studies have shown OPCAB to be associated with a lower incidence of stroke compared with that using conventional on-pump CABG [13, 14]. Particularly in high risk patients, such as those with cerebrovascular atherosclerosis, severe aortic atherosclerotic, or peripheral artery disease, OPCAB reduced stroke incidence [2, 15]. Therefore, we selected OPCAB for this patient. Furthermore, we applied IABP to minimize hypotension and decreased cardiac output during displacement of the heart. IABP is acknowledged to maintain cardiac function [16], and to improve CBF, particularly in patients with preexisting heart failure, highly impaired left ventricular ejection fraction [17], and severe CIAD [18]. In animal study, augmentation of cardiac output and pulse pressure by IABC results in an increase in local CBF in ischemic brain [19].

## Conclusions

Preoperative quantitative evaluation using brain perfusion SPECT with acetazolamide is useful in assessing hemodynamic cerebrovascular risk in patients with severe obstructive CIAD. OPCAB with IABP assist is a good option for prevention of cerebrovascular morbidity in ischemic heart disease with severe CIAD.

## Data Availability

The data is available from corresponding author on reasonable request.

## Abbreviations

123I-IMP: N-isopropyl-p-[123I]iodoamphetamine
CABG: Coronary artery bypass grafting
CBF: Cerebral blood flow
CIAD: Carotid and intracranial artery stenotic and/or occlusive disease
ICA: Internal carotid artery
MCA: Middle cerebral artery
MRI: Magnetic resonance imaging
OPCAB: Off-pump coronary artery bypass grafting
rCBF: Reserve cerebral blood flow
rCBR: Reserve cerebral blood response
SPECT: Single-photon emission computed tomography

## References

[1] Naylor AR, Mehta Z, Rothwell PM, Bell PRF (2002). Carotid artery disease and stroke during coronary artery bypass: a critical review of the literature. Eur J Vasc Endovasc Surg.

[2] Tsuda K, Shiiya N, Washiyama N, Yamashita K, Ohkuma K, Takahashi D, Kando Y, Natsume K, Yamanaka K, Takeuchi Y (2018). Carotid stenosis with impaired brain flow reserve is associated with an increased risk of stroke in on-pump cardiovascular surgery. Interact Cardiovasc Thorac Surg.

[3] Kuroda S, Houkin K, Kamiyama H, Mitsumori K, Iwasaki Y, Abe H (2001). Long-term prognosis of medically treated patients with internal carotid or middle cerebral artery occlusion: can acetazolamide test predict it?. Stroke.

[4] Imasaka K, Yasaka M, Tayama E, Tomita Y (2015). Obstructive carotid and/or intracranial artery disease rarely affects the incidence of haemodynamic ischemic stroke during cardiac surgery: a study on brain perfusion single-photon emission computed tomography with acetazolamide. Eur J Cardiothorac Surg.

[5] Imasaka K, Tayama E, Morita S, Tomita Y (2018). Neurological outcome and efficacy of intensive craniocervical screening for elective cardiac surgery. Interact Cardiovasc Thorac Surg.

[6] Yamauchi H, Fukuyama H, Nagahama Y, Nabatame H, Nakamura K, Yamamoto Y (1996). Evidence of misery perfusion and risk for recurrent stroke in major cerebral arterial occlusive disease from PET. J Neurol Neurosurg Psychiatry.

[7] Mizumura S, Nakagawara J, Takahashi M, Kumita S, Cho K, Nakajo H, Toba M, Kumazaki T (2004). Three-dimensional display in staging hemodynamic brain ischemia for JET study: objective evaluation using SEE analysis and 3D-SSP display. Ann Nucl Med.

[8] Caplan LR, Hennerici M (1998). Impaired clearance of emboli (washout) is an important link between hypoperfusion, embolism, and ischemic stroke. Arch Neurol.

[9] Silvestrini M, Vernieri F, Pasqualetti P, Matteis M, Passarelli F, Troisi E, Caltagirone C (2000). Impaired cerebral vasoreactivity and risk of stroke in patients with asymp- tomatic carotid artery stenosis. JAMA.

[10] Lamy A, Devereaux PJ, Prabhakaran D, Taggart DP, Hu S, Paolasso E, Straka Z, Piegas LS, Akar AR, Jain AR, Noiseux N, Padmanabhan C, Bahamondes JC, Novick RJ, Vaijyanath P, Reddy SK, Tao L, Olavegogeascoechea PA, Airan B, Sulling TA, Whitlock RP, Ou Y, Pogue J, Chrolavicius S, Yusuf S (2013). Effects of off-pump and on-pump coronary- artery bypass grafting at 1 year. N Engl J Med.

[11] Diegeler A, Börgermann J, Kappert U, Breuer M, Böning A, Ursulescu A, Rastan A, Holzhey D, Treede H, Rieß FC, Veeckmann P, Asfoor A, Reents W, Zacher M, Hilker M, GOPCABE Study Group (2013). Off-pump versus on-pump coronary-artery bypass grafting in elderly patients. N Engl J Med.

[12] Shroyer AL, Grover FL, Hattler B, Collins JF, McDonald GO, Kozora E, Lucke JC, Baltz JH, Novitzky D (2009). On-pump versus off-pump coronary-artery bypass surgery. N Engl J Med.

[13] Nishiyama K, Horiguchi M, Shizuta S, Doi T, Ehara N, Tanuguchi R, Haruna Y, Nakagawa Y, Furukawa Y, Fukushima M, Kita T, Kimura T (2009). Temporal pattern of strokes after on-pump and off- pump coronary artery bypass graft surgery. Ann Thorac Surg.

[14] Puskas JD, Kilgo PD, Lattouf OM, Thourani VH, Cooper WA, Vassiliades TA, Chen EP, Vega JD, Guyton RA (2008). Off-pump coronary bypass provides reduced mortality and morbidity and equivalent 10-year survival. Ann Thorac Surg.

[15] Doi K, Yaku H (2010). Importance of cerebral artery risk evaluation before off-pump coronary artery bypass grafting to avoid perioperative stroke. Eur J Cardiothorac Surg.

[16] Kim KB, Lim C, Ahn H, Yang JK (2001). Intraaortic balloon pump therapy facilitates posterior vessel off-pump coronary artery bypass grafting in high-risk patients. Ann Thorac Surg.

[17] Pfluecke C, Christoph M, Kolschmann S, Tarnowski D, Forkmann M, Jellinghaus S, Poitz DM, Wunderlich PC, Strasser RH, Schoen S, Ibrahim K (2014). Intra-aortic balloon pump (IABP) counterpulsation improves cerebral perfusion in patients with decreased left ventricular function. Perfusion.

[18] Tsukube T, Okada M, Ataka K, Ozaki N (2001). Coronary artery bypass grafting in a patient with brain ischemia. J Cardiovasc Surg.

[19] Tranmer BI, Peniston C, Iacobacci R, Salerno TA, Hudson AR (1989). Intra-aortic balloon counterpulsation: a treatment for Ischaemic stroke?. Neurol Res.

